# Ten simple rules for building a successful science start-up

**DOI:** 10.1371/journal.pcbi.1009982

**Published:** 2022-04-07

**Authors:** Tobias Reichmuth, Collin Y. Ewald

**Affiliations:** 1 Maximon AG, Zug, Switzerland; 2 Laboratory of Extracellular Matrix Regeneration, Institute of Translational Medicine, Department of Health Sciences and Technology, Swiss Federal Institute of Technology (ETH Zürich), Schwerzenbach, Switzerland

## Overview

Certain rules do apply when building a start-up company. The core principles of these rules are fundamental across industries, including academia. The following 10 rules guide academic researchers and business-trained entrepreneurs who want to take a leap of faith to adopt the recent advances in sciences for commercialization. These rules provide an entry point and raise challenges to be thought about for a starting entrepreneur. We believe that these rules apply to any innovation-based start-up and help prioritize the most impactful tasks. Understanding these rules will help the founding team, investors, and other stakeholders to launch a successful start-up.

## Introduction

Each start-up addresses a specific problem to which the solution is of value to the customer. While building a company can be fun and rewarding, at each step in developing this solution, there are decisions to be made that have consequences that either lead to failure or success. Some mistakes made early in this journey can be fatal and are hard to correct. Therefore, a founder must think about many issues to mitigate potential pitfalls. The complexity, the different constraints, the dynamic landscape of this journey, and practical step-by-step how-to advice are beyond this 10 simple rule format. They are captured in several excellent books [1–4] written to address many obstacles on this journey, which are referred to in the specific rules. However, while there is no one-size-fits-all advice, the rules introduced in this paper are general and apply to most founders.

There are several important topics for academic founders that are relevant and must be addressed before founding a science-based company:

1. University
  - What are the university’s constraints for a start-up led by a student, postdoc, or faculty? Some universities allow 20% of the employee’s time to take on an operational role in a start-up for some time, while others might not be accommodating for extra activities.
  - If you stay as a faculty, how do you manage conflict of interests? Are you allowed to be a board member of the company?
2. Intellectual property (IP)
  - Have you filed a patent application? Usually, the IP or patent stays at the university and is licensed out. Have you talked to your tech transfer office about the licensing terms? Do you get exclusive rights to your patent? What are the licensing fees? Or do you want to keep your knowledge as a trade secret to be sold to your company? If so, did you disclose everything to your tech transfer office?
  - Do you have the freedom to operate? Is there a patent on a procedure that would block you from building your product or commercialization?
  - For e-commerce or software-based companies, do you distribute your software under an open-source license, or do you want/need to license it?
3. General business knowledge
  - As an academic, do you understand the business aspects, and are you familiar with the start-up culture?
  - Are you aware of legal company structures and equity dilution versus capital raised?

To address these questions, universities provide courses and practical workshops for students. Several online courses cover specific details and technical hurdles to transferring technology or knowledge from academia to a start-up. Beneficial are classes on how to write a business plan or develop and pitch an idea, and we encourage researchers to take up such offers.

With this 10 simple rules paper, we take a step back and provide an overview of challenges known by serial entrepreneurs, venture capitalists, and investors that are, in our view, essential but likely would not have been considered by an academically trained person. The 10 rules are not meant as a step-by-step guide, and they are not exhaustive. Although we start with the most common misconceptions, the listed order of the 10 rules does not necessarily constitute relevance. A limitation of our proposed rules is that since we aimed to capture the core principles that would apply to a broad range of company settings, they do not include several specific and fundamental issues that arise, e.g., for biotech versus e-commerce start-ups. Thus, the rules are more conceptual and abstract but central for innovation-driven start-ups. In each rule, we briefly provide more specific issues, questions, and advice for the founders to be aware of and overcome potential challenges. As Louis Pasteur once pointed out, “… chance favors only the prepared mind” [5].

### Rule 1: The team is more important than the idea

Some words to start with: This rule might seem counterintuitive for a scientist. An idea could be a novel hypothesis to explain a set of observations and then use this idea to determine the underlying mechanism(s). Here, by an idea, we refer to an invention or technology, service or product, or some sort of IP. As an innovation-driven entrepreneur, you aim to commercialize your invention. Innovation can be defined as invention multiplied by commercialization [1]. If you have an invention but zero commercialization, the end product is zero. Similarly, there will be no end product if you have no invention but commercialization. Thus, to bring your idea to the market, you need a highly effective team to develop your innovation into a product wanted by your customers.

Given that you have secured unique technical knowledge either as patents (own IP or exclusive rights) or as trade secrets, building a start-up is not a straightforward exercise. It needs constant adaptation, sometimes pivoting from the original business plan. This asks for a great deal of flexibility from the founders: Original assumptions may be changed, new tasks and responsibilities may arise, and the duties and priorities of the team members can change. Due to the initial budget constraints—a reality for pretty much all start-ups—tasks cannot be delegated but must be taken on by the founders. Therefore, the founding team must represent a diversified set of qualifications, not limited to scientific know-how, but also business know-how, sales spirit, manager qualities, and industry knowledge. Based on the authors’ experience, the only thing that can be delegated is the legal aspect. It is essential to have legal expertise available; however, a start-up budget cannot typically finance an in-house lawyer. Moreover, lawyers might be too much of a devil’s advocate. A founding team needs to focus on why things can work and not why they might not.

One key challenge is that the mindset of technical versus business founders is entirely different [3]. A scientist is trained to share his/her discoveries in peer-reviewed journals, drive innovation, improve technology, and work on novel and unique aspects of a given problem. By contrast, the business driver tries to keep the research focused on the commercialization of the core technology [3] or does not invest too much time in research but follows his/her gut feeling or copies from other business models. The value system and ethics of scientists and business drivers often are different. Generalizing, one can say that scientists care about what their peers think—i.e., the impact of their latest discovery. By contrast, business drivers care about bringing the technology to the market to make money. Thus, frictions in mixed founder teams can stem from the clash of 2 cultures. If founders have not experienced the 2 different worlds, it becomes crucial to understand the motive and thinking of the other “point of view.” Proper conversations and frequent communications are vital.

The founders often work long hours and spend a lot of time together: It is, therefore, vital that team members embrace different viewpoints and respect each other. Similar to a marriage, it will need compromises, and not so different from marriage, it also might not work out. In comparison, this should be avoided by carefully selecting your cofounders preparing for such a scenario. Like a marriage agreement, which protects the partnering individuals and potential offspring, a partnership or shareholders’ agreement to safeguard the company’s interests is a must (here, we suggest consulting a start-up experienced law firm). In this context, it is vital to avoid a potential deadlock situation: Should the founding team consist of only 2 founders, a 50/50 shareholding might represent a danger for the company. Therefore, we advise defining clear rules on decision finding or avoiding the 50/50 shareholding situation.

The next critical point for forming a founding team is the individual founder’s long-term motivation: Why are you building a company? Is a fast exit the goal or rather the formation of a market leader over the next 10 to 20 years? Do you want to solve a problem for society or the planet, or is making money your main driving force? Whatever it is, the team members must be aligned with the time horizon when they embark on their venture.

In summary, it is easy to understand that building a founding team is no trivial task. The following parameters are therefore key when building a great founding team:

- Heterogenous background: Team members should complement each other and bring all needed skills together. A theory coined by Rei Inamoto (AKQA) [6] posits that for a successful science start-up, you need the “3H” team-building strategy for founders: a hipster, a hacker, and a hustler [7]. The hipster—the chief marketing officer (CMO)—is the creative genius who adopts the technology to make it cool and appealing (i.e., covers design and user experience). The hacker or nerd—the chief technology officer (CTO)—is the brain behind the technology and creating the actual product (i.e., covers technology engineering and developing). The hustler—the chief executive officer (CEO)—moves the project forward, makes sure to hold the course with the vision, builds awareness and a network, is key to selling the product, and, of course, raises the necessary funds (i.e., covers finances, marketing, and business).
  Note for academics. Usually, science-based start-ups are founded by PhD students at the university, consisting of the researchers involved in the science and the professor, who developed the technology and generated the IP. Although, as an intelligent scientist, you can learn all about running a business, the business mindset will not come naturally to you, like learning a new language as an adult. Thus, a team consisting of only scientists might fail without an entrepreneur with profound business training. Furthermore, it is essential to learn how the business mindset approaches the same problems to prevent miscommunication.
- Homogenous motivation: Team members should have a similar long-term reason for building the company and, more importantly, share the same vision and time horizon.
- Partnership or shareholders’ agreement: While founding team members must get along well, it is necessary to have a partnership or shareholders’ agreement in place, safeguarding the company’s interests.
- A 50/50 shareholding should be avoided, or clear decision-finding rules must be defined upfront in case of a deadlock.

Thus, a great team can succeed with a mediocre business idea, while a mediocre team will fail even with a great business idea.

### Rule 2: Hire slow, fire fast

It is hard to sack people. This is even harder if they have become friends and given their best efforts (which was not good enough). How can this be avoided, and why does it have to be done fast if the situation arises?

Hiring the right talent is key to the success of any company, but even more so for a start-up: In a start-up setting, you simply don’t have enough time to work with the wrong talent. There should be no redundancies, and every team member must work independently. A team is performing only and its weakest member [8]. As a founder, it is critical for your company’s success to spend enough time recruiting the right talent.

Even if you did invest a lot of time in recruiting the right talent, inevitably, at some point, some team members will not live up to their tasks. As the company grows, responsibilities grow. A team member who might have been a perfect fit in leading a 3-person team might lose the oversight when managing 30 team members. It is then crucial to act fast: Either you find a new and suitable position for this person or you must say “goodbye.”

In academia, this is a familiar situation: One of the most common beginner mistakes as a newly appointed group leader is to ignore the red flags indicating that the PhD student or postdoc is not up to the challenge [9]. After providing support and help, it is hard to let people go, mainly since such decisions are based on a few indicators, like the decline in performance and increased stress levels. But acting fast is vital. Stressed-out lab members will negatively affect and stress out other lab members (i.e., like a dominant-negative mutation) and bring down the entire team’s mood, optimism, performance, and drive.

Similarly, an entrepreneur must follow his/her gut feeling: If there are signs that a team member is not up to the task, then this is probably the case. Do not wait to resolve this situation. You will not do this person a favor by keeping him/her longer in this position or the organization—he/she will do better in a new place tailored to his/her skills. Hence, the wrong person for the job will slow the company down, and time is of the essence in a start-up environment. In short, hire slow, fire fast.

### Rule 3: It costs more and takes longer

Anyone who has ever planned and taken on a science project will undoubtedly experience that it always takes longer than planned. No matter how elegant, simple, and straightforward the well thought through research grant proposal looks on paper, breaking new research grounds is more complicated in reality. Similar to a research proposal, writing a business plan is an excellent exercise for founders. Next to being a persuading tool in getting team members and investors on board, it helps to understand dependencies, set priorities, and provide a rough idea of the resources needed to build the company. However, compared to a research proposal, the complexity of the moving parts in a business plan is significantly higher, and, therefore, the capital needed is more difficult to establish.

Most companies fail not because the idea is inherently bad but because they run out of funds [3]. Having enough capital is therefore critical to overcoming the “Valley of Death” (i.e., from the initial capital to generating revenues [10]) and becoming a successful market leader.

As a founder, you aim to give away as few shares as possible while at the same time securing the necessary capital to build the company. Two aspects are of importance:

- Various financing rounds are the norm. It is unnecessary to secure all the financing up to break even on day 1, as this would dilute the founders too much. Instead, it is advisable to split the capital needs based on the company’s current stage. When milestones are reached, the company’s valuation can be increased, and additional capital can be raised at a minor dilution.
- At the same time, the founding team should not be distracted by constant fundraising but instead should focus on product development. It makes sense to raise enough capital (i.e., more than you think is needed) to deal with delays in the future.

Balancing these 2 aspects—the wish for a minimal dilution and the need for sufficient financing—is not an easy exercise. Do not raise too much money in the beginning. However, the following has universal truth as a guiding rule for seed rounds: It costs more and takes longer. We, therefore, suggest raising double the amount you think is needed—at least in your first seeding round. Furthermore, it is unlikely that things work the first time perfectly, so set your timeline to have at least 12 to 18 months of buffer time that is financially secure for each milestone.

### Rule 4: A unique business idea is not required

Many founders believe that a start-up company can only be successful if the business idea is unique. This misconception is hard to understand as a scientist, where uniqueness and novelty are key for scientific success. Of course, for patenting your technology, it has to be nonobvious to prior knowledge [11]. You need unique technical knowledge either as patents (own IP or exclusive rights) or as trade secrets to set your science start-up apart from competitors (i.e., IP is central for ensuring competitive advantage). Furthermore, your idea needs to meet the real-win-worth (R-W-W) criteria [12]: Is it real? Can we win? Is it worth doing? However, the uniqueness of the business idea is less crucial. Peter Thiel classified start-ups into 2 groups: from 0 to 1 (creating something new) or from 1 to *n* (adding more of something familiar) [2]. Most successful companies were seldom the first ones in the market (e.g., Google was not the first search engine, Tesla was not the first electric car producer, and Apple Macintosh was not the first personal computer). The first company introducing a new service or product must pave the way: It takes a certain effort to explain to the general audience that a new solution for a (maybe not even yet known) problem exists. Surfing the wave of a newly emerging market that just started to grow might be the easier way to succeed. Often very successful companies are copycats from abroad: a business idea that works in the United States of America or Japan might work in Europe. Copying a business idea represents, therefore, risk reduction. If you copy a business idea in the same markets as your competitors are already active in, this market either needs to grow fast or you need to have a better approach to serve the customers’ needs. That said, it might be easier to find investors for a venture that is based on an already successful business idea from another market. From that perspective, the competition allows for a certain validation of a business idea.

### Rule 5: Better share than stealth

Many founders think that their business idea is unique and nothing short of ingenious. They, therefore, might think that talking about their business idea is risky: The idea might be copied or, even worse, stolen by an evil and, of course, financially powerful conglomerate. With this in mind, they do not talk about their business idea, and if they do, they ask to sign a nondisclosure agreement (NDA) firsthand. They forego many potential opportunities with this: Nobody is aware of what the founders are working on and cannot offer advice. Maybe other teams have worked on the business idea and found it impossible to succeed for structural reasons? Perhaps the founder’s network could provide connections to much sought-after team members or investors?

No professional investor will sign an NDA before reaching a general understanding of the business idea (it would simply constrict an investor’s future activities too much). In general, it is highly unlikely that another entrepreneur has (a) the know-how, (b) the time, and (c) the resources to copy (steal) a business idea. More likely, such a person would want to join the venture or at least offer critical feedback that might help improve the business idea. Therefore, we advise you to talk about your business idea with as many people as possible and execute it quickly. You will get support from many unexpected sides, and your business idea will be sharpened and optimized on the way. As in science, discussing the “killer” experiment with your peers to disproof your hypothesis, it is important to identify this weak spot that could collapse your idea, like a house of cards. Hence, if you fail, fail fast. Moreover, as you strive to derisk your technology in the early stages of commercialization, bouncing back your business ideas with others helps to derisk and improve your path forward with your business idea.

In practice, you have 2 pitch decks: a confidential and a nonconfidential version. The nonconfidential pitch deck states the importance of the problem and how you are able to solve it. This pitch deck indicates the market and customer you will address and covers the conceptual idea without any technical secret but highlights your competitive advantage. With the confidential pitch deck that you share under an NDA, you will dive more into the nitty-gritty of your proposed idea/solution and can share data. Make sure you know your audience to adjust these pitch decks targeted to the audience interest/expertise (e.g., more technical versus financial).

### Rule 6: Focus, focus, focus (on time to market)

Start-up entrepreneurs usually are short of everything: time, money, and personal resources. It is, therefore, of existential importance to generate returns as early as possible. In other words, it is essential to reduce the “time to market,” i.e., the time span between the foundation of a company and its first turnover generated by making its product available to customers. This can be reached by one elementary exercise: focus.

Similar to your success in science that depends on your publication output, i.e., the “publish or perish” principle, a founder team should have only one focus: how to bring the product to the market or customer—fast. This is hard to internalize by researchers who do research for the sake of research [3]. It is crucial to focus only on the research necessary to generate the pilot [3], i.e., no overengineering is needed. The general path for technology-driven ideas is first to have the proof-of-concept in the form of a prototype (i.e., the technology works in the lab), then transfer the technology from lab equipment to commercial equipment (i.e., the pilot). Hence, “start with the end in mind” [13] and then work yourself backward to outline the most critical steps required for the shortest path to develop your idea into the pilot, derisk the technology, develop up-scaling and manufacturing capabilities, and bring it to the market. For a biotech or pharmaceutical start-up developing drug candidates (either derived from living organisms or on a chemical basis, respectively), your customer is likely to be a bigger pharma company. Your goal is to minimize the time it takes to develop your drug candidates to Phase I or II clinical trials. The same principle applies: FOCUS on pushing your candidate through your drug development pipeline and presenting a so-called minimal viable product (MVP).

As explained in Rule 3 above, it is essential not to be distracted by constant fundraising. Neither should too much time be invested in participating in business plan contests—you are not successful by winning awards, but by generating revenues! This also means that many other topics must be pushed back: The company’s brand name is not worth spending weeks on. As long as the name has no negative connotations, you can name, e.g., a computer company after a fruit and be successful with it. Moreover, neither should too much time be spent on logos and corporate identity nor on the furnishing of your office. The focus for the first 2 to 3 years is the product or service, which also must not be overengineered. The MVP is what you should bring to market fast. Aim for the 80/20, the Pareto principle [14]. Identify the 20% of activities that result in 80% of the desired outcome [15]. Once you know what to focus on, such as how to get the minimum viable product, set short deadlines to execute the plan according to Parkinson’s law [15]. Based on the customers’ reactions, you can then improve the product and enhance your design and other features.

### Rule 7: Customers do not tell you—you need to analyze their needs

Market research is generally a good idea; however, you cannot expect your future customers to tell you how your product should look or how it should perform. As Henry Ford is famously quoted for, “If I had asked people what they wanted, they would have said faster horses” [16]. As an entrepreneur, it is not necessarily a must to do extensive surveys, but rather to identify problems and needs and quickly come up with an MVP and test it in the market [4]. Analyzing the issues people suffer from can provide excellent insights into the potential market size for your product. As for developing a drug, you need to know which disease you address and which indication to target. It is essential to segment the market and identify the targeted customer groups for your solution. Getting feedback from these initially identified customers on whether a solution is wanted and of value is essential [1]. However, once you have defined your product, it does make sense to ask customers for their opinion. This ensures that you do not (over) engineer your product away from market needs while fulfilling the regulatory standards. The situation is not different in a research-based start-up: The general audience is typically not informed well enough to bring suggestions for new products and services to the table.

### Rule 8: No dead equity

Founders feel great upon securing the first financing round for a company. People entrusting capital at risk to you and allowing you to build a company is an important milestone. Mainly, those early investors are recruited from the entrepreneur’s existing network. These investors qualify as the “Triple-F-Investors,” i.e., Family, Friends, and Fools. It is questionable, though, whether this type of investor is helpful or—at least—not a burden for the development of your company.

Triple-F-Investors typically do not know much about your industry. Most of them will be quietly proud of what you achieve, but they cannot help you build the company. However, while they might be quietly observing initially, there is a risk that they start to be protective about their investment once the company generates the first successes. Later, these investors can begin to show more interest in your daily business than would be healthy. While they do not understand your industry and the company’s needs, they want to have a say over the strategy or even the operational management of the start-up because they fear for their now much more valuable investment. This can lead to problems and can slow down the firm’s development.

It is, therefore, essential to invest some time in the structure of your capitalization (cap) table. A cap table is a spreadsheet showing the distribution of ownership of the company, the equity dilution, and the value of equity for each financing round. It tracks the company’s equity ownership by the owners, founders, and investors. A note of caution for academic founders to manage expectations: Usually, investors rebalance your proposed cap table. Furthermore, you should be asking the following: What qualifications must investors bring along with their money? Is the investor helpful to envision your company’s future? At what stage is which experience and network useful? Will at least some investors from your seed round be able to invest in future rounds? If no existing investor is investing in future rounds, it might be hard to raise additional capital from new investors. Thus, you grant your investors the chance to invest in something that potentially becomes big—while you should be thankful that they entrust their money to you, you can also be asking for other relevant input.

As a general rule, avoid “dead equity,” never grant board seats to not savvy minority investors, and make sure that your early investors can both invest in future rounds and help the company with their experience and network.

### Rule 9: Modesty comes later

Modesty is a good trait. However, while it is noble to be modest once you are successful, it is not the first thing you should think about when building a company. Academics are usually cautious with their statements and avoid hyperbole of their scientific findings or idea. You need to learn how to sell your idea. Do not shy away from telling the world why you are not just different but superior. Immediately say what your unique twist is. Investors and customers alike must hear about you: being present in the relevant media, being talked about, etc. These are essential assets when building a company. A steady stream of good news will achieve this. Having good news can prove difficult when you create a start-up, which also involves suffering from setbacks. However, there is always something positive to talk about: outstanding new team members, a successful product test, an article about your company, or the last team event you have organized. Make sure people hear of you and never hold back with positive news. If you have to communicate something positive, do it now! There is no reason to wait, and it does not make sense to gather good news and share them all at once. It is much more effective to repeatedly have good news, which will anchor you in people’s memory. While there is the familiar quotation “fake it till you make it,” you should never communicate something that is not true or nonfact based. However, it is helpful to adopt the most positive wording to describe a new achievement in the early days of your company.

### Rule 10: Credibility via advisory board

Why should customers (and investors) trust you that you really can build the product you plan to? And are you sustainable for the long term? It might be difficult for customers—especially for business-to-business (B2B) customers—to build a business relationship with you if they cannot be sure that you are still around some months from now. How to overcome this?

One effective and not too costly way is to install a top-notch advisory board. For a biotech start-up, ask Nobel prize winners, national science academy members, previously successful veteran entrepreneurs, and professors providing core expertise to complement your team. There is a ranking of scientific advisory board members, probably in the order, as we had listed them above. For a business advisory board, ask seasoned industry veterans to support you publicly by appearing on your homepage and by giving their advice to you. While this can seriously improve your company, it also sends a strong signal to your customers and investors. If this highly regarded professional has joined your advisory board, then certainly there must be something worthwhile about your company.

Usually, it is not difficult to recruit a great advisory board—successful industry veterans might like the idea of supporting a dynamic start-up, and they might even learn something they can use in their careers. Similarly, ask professors and experts with technical knowledge and expertise in areas your company lacks. As a rule of thumb, you can attribute around 0.5% to 1% of your shares to a top-notch advisor over 3 years, depending on what value they bring to your company (time commitment sharing their expertise, network, access to capital, etc.). In many cases, it is worth doing.

## Conclusive remarks and perspectives

Many factors contribute to the success of building a start-up. Based on our experience, we identified some common patterns that we incorporated into 10 simple rules. We think these rules are the 20% that covers 80% of the success. However, simply applying these rules does not guarantee success. Take these rules as direction or advice. As applies to many things, too much or too little of a good thing is bad. The key is to find the right balance. Understanding the concepts and principles of these 10 rules will help entrepreneurs avoid mistakes commonly made by first-time entrepreneurs. As the last suggestion—if we may—we advise you not to wait too long with founding your company. Your opportunity cost will only rise, and the decision to start a company will become more challenging to take. The best moment to become an entrepreneur is now!
